# Shunt infusion studies: impact on patient outcome, including health economics

**DOI:** 10.1007/s00701-020-04212-0

**Published:** 2020-02-20

**Authors:** Afroditi-Despina Lalou, Marek Czosnyka, Matthew R. Garnett, Eva Nabbanja, Gianpaolo Petrella, Peter J. Hutchinson, John D. Pickard, Zofia Czosnyka

**Affiliations:** 1 Department of Clinical Neurosciences, Division of Neurosurgery, Cambridge University Hospital NHS Foundation Trust, Cambridge, UK; 2 Department of Neurosurgery, Ospedale S.M. Goretti, Latina, Italy

**Keywords:** Cerebrospinal fluid diversion, Hydrocephalus, Idiopathic intracranial hypertension, Infusion studies, Pseudotumour cerebri, Shunts, Shunt testing in vivo

## Abstract

**Objectives:**

The diagnosis of shunt malfunction is often not straightforward. We have explored, in symptomatic shunted patients with hydrocephalus or pseudotumour cerebri syndrome (PTCS), the accuracy of CSF infusion tests in differentiating a functioning shunt from one with possible problems, and the health economic consequences.

**Methods:**

Participants: hydrocephalus/PTCS patients with infusion tests performed from January 2013 until December 2015. We followed patients up after 6 and 12 months from the test to determine whether they had improved, had persisting symptoms or had required urgent revision. We calculated the total cost savings of revision versus infusion tests and standard protocol of revision and ICP monitoring versus infusion tests.

**Results:**

Three hundred sixty-five shunt infusion tests had been performed where a shunt prechamber/reservoir was present. For hydrocephalus patients, more than half of the tests (~ 55%, 155 out of 280) showed no shunt malfunction versus 125 with possible malfunction (ages 4 months to 90 years old). For PTCS patients aged 10 to 77 years old, 47 had possible problems and 38 no indication for shunt malfunction. Overall, > 290 unnecessary revisions were avoided over 3 years’ time. Two hundred fifty-eight (> 85%) of those non-surgically managed, remained well, did not deteriorate and did not require surgery. No infections were associated with infusion studies. For Cambridge, the overall savings from avoiding revisions was £945,415 annually.

**Conclusions:**

Our results provide evidence of the importance of shunt testing in vivo to confirm shunt malfunction. Avoiding unnecessary shunt revisions carries a strong health benefit for patients that also translates to a significant financial benefit for the National Health Service and potentially for other healthcare systems worldwide.

**Electronic supplementary material:**

The online version of this article (10.1007/s00701-020-04212-0) contains supplementary material, which is available to authorized users.

## Introduction

The most recent epidemiological study from the UK Shunt Registry demonstrates that 20% of CSF shunts fail within 1 year of primary insertion, ranging from 31% in infants to 17.4% in adults (Fernandez-Mendez R et al. 2019, in press). The main reasons for revision were underdrainage and infection, but overdrainage and mechanical failure continue to pose problems. The diagnosis of shunt malfunction based on a careful clinical history, examination, and investigations such as computed tomography (CT) scanning and plain X-ray shunt series, is not always straightforward [45]. For example, ventricular size may not change in cases with a blocked shunt. Pumping a shunt prechamber is notoriously unreliable and potentially dangerous [5]. Admission for observation is expensive and excessive CT scanning carries a radiation burden. Many patients may be admitted and subjected to CT scanning on multiple occasions. There is a need to develop more reliable methods of assessing shunt function and monitoring intracranial pressure (ICP) [18, 27, 36, 42, 44, 46]. Intrashunt injection of a radionuclide, or creation of ultrasound bubbles or a thermal gradient all have their advocates. Optic nerve sheath diameter may be assessed using ultrasound or magnetic resonance imaging (MRI). Implantable ICP sensors within a shunt system have been blighted by poor long-term stability. Long-term studies of the recently introduced Raumedic Neurovent P-tel and the Miethke prechamber ICP sensor are awaited with keen interest [1].

Non-invasive techniques to assess ‘semi-quantitatively’ whether intracranial pressure is raised or not include optic nerve sheath diameter (ultrasound or MRI), tympanic membrane displacement and transcranial Doppler but none have yet been shown to be sufficiently accurate for routine clinical use in patients with potential shunt malfunction.

Provision of a separate subcutaneous CSF reservoir is of proven benefit in allowing access to the cerebral ventricles to measure ICP and allow removal of CSF in an emergency [30]. CSF infusion tests are an elaboration of the tap-test that were introduced into clinical practice over 45 years ago and have been reported to be a minimally invasive, low risk and potentially useful diagnostic tool for testing shunt function in vivo [10, 13, 20, 21, 31]. However, they still lack widespread endorsement. In this study, we aim to show that shunt infusion tests are accurate in terms of differentiating between a working shunt and a shunt with complete or partial failure such as under-, overdrainage or blockage [6, 14, 37, 47], aid resetting of adjustable valves, help refine shunt revision surgery and are cost-effective by avoiding unnecessary admissions and revisions.

## Materials and methods

### Patient data

We identified and collected the results of all shunt infusion tests performed between January 2013 and December 2015. These included a total of 280 computerised CSF infusion tests which were performed in 210 shunted hydrocephalus patients of all ages and aetiologies. As part of our routine clinical pathway, all shunted individuals presenting with sudden onset, clear symptoms of raised ICP and an unequivocal CT head would have had an urgent revision and therefore do not require a shunt infusion test.

Besides the hydrocephalus shunt infusions, we included 85 tests on PTCS patients (including idiopathic intracranial hypertension (IIH)) and analysed them separately because of differences and more complexities compared to hydrocephalus patients. However, the shunts implanted in those patients do not differ significantly in properties and the same principles of shunt testing in vivo apply to them as well, as infusion objectively tests the hydrodynamic properties of the implanted shunt.

### Shunt infusion test procedure and result interpretation

Infusion test results and conclusion on the shunt’s function had been reported independently by a clinical physicist at the time of request and the conclusion statement (blocked, under-/overdraining and properly functioning) was used to match against clinical outcome.

Objective testing of implanted shunts through infusion studies is a well-refined method based on both in vitro and in vivo knowledge of all marketed shunts’ hydrodynamics, primarily the shunt critical pressure and its resistance [6, 10, 13]. Validation of the methodology in relationship to clinical and intraoperative findings of shunt revision has recently been performed in a large paediatric population from two European centres [17].

**Fig. 1:**
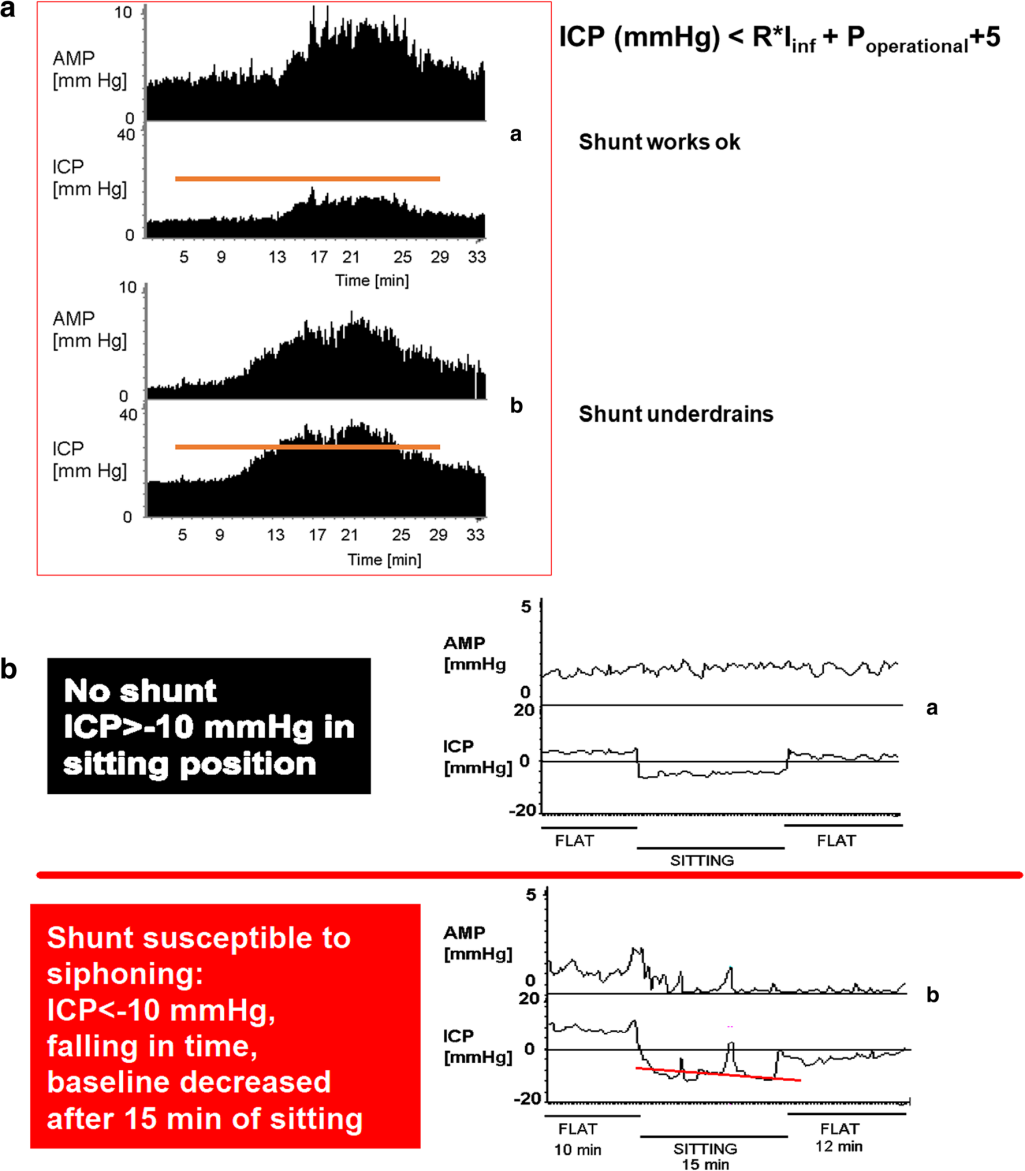
Shunt testing results of under- and overdrainage. **a** Top: Normally functioning shunt, with the plateau (steady-state) pressure after infusion of Hartmann’s not exceeding the shunt’s critical ICP. Bottom: Underdraining shunt, whereby the critical ICP is exceeded by a few mmHg. The formula at the top explains how the critical ICP is calculated. A clearly detected ICP pulsation and amplitude (AMP) confirms the presence of communication with the ventricles, and therefore a patent ventricular catheter. All calculated parameters are derived from the UK shunt laboratory. **b** Slow phase response of decrease in ICP in an individual without a shunt versus fast response of decrease in ICP to < − 10 mmHg in a shunt with antisyphon device failure causing posture-related overdrainage. After laying flat again, ICP is significantly lower than the original baseline. ICP intracranial pressure, AMP amplitude of intracranial pressure. Figure modified with permission from Czosnyka, Zofia, Czosnyka, Marek, Pickard J. Shunt testing in vivo: A method based on the data from the UK Shunt Evaluation Laboratory. *Acta Neurochir Suppl*. 2002; 81:27–30

**Fig. 2:**
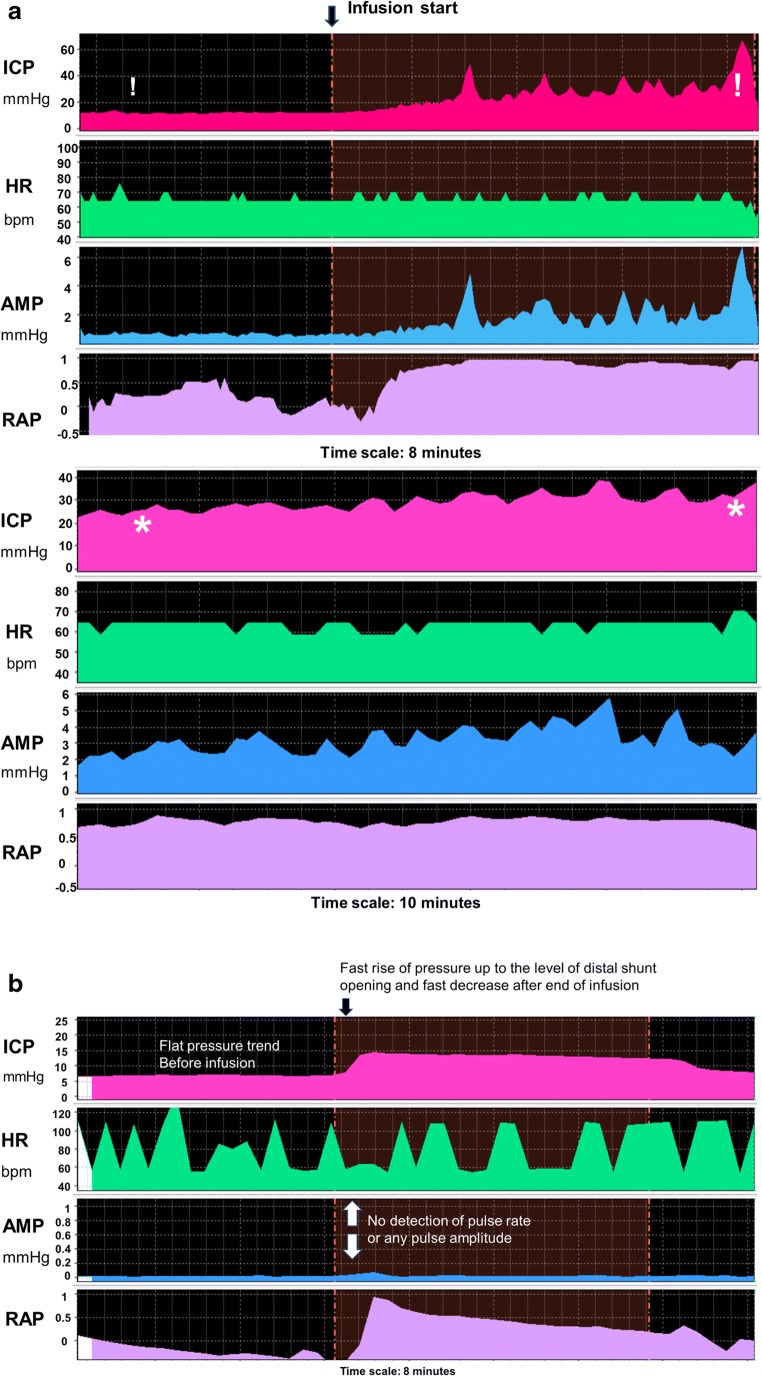
Shunt testing results of proximal and distal obstruction. **a** Distal obstruction. Upper panel: distal obstruction detected after infusion of fluid. Initial baseline ICP appears normal (c. 12 mmHg); however, ICP increases to > 25 and towards the end to > 40 mmHg, completely out of range for a functioning distal catheter. Lowe panel: distal obstruction evident from initial monitoring of baseline ICP for 15 min. The opening ICP was > 20 mmHg, spontaneously increasing to > 30 mmHg after 5 min of monitoring, revealing sever intracranial hypertension caused by a patent ventricular, however blocked distal catheter. Infusion was not performed as it is unsafe in such high ICP. **b** Proximal (ventricular catheter) obstruction as evidenced by a lack of pulsation and therefore heart rate and amplitude detection from the pressure inside the shunt prechamber. This demonstrates lack of connection with the ventricles. Infusion can be started as normal, and an unobstructed distal run-off is detected through stabilisation of pressure at the expected critical level. All 3 patients had no or very mild changes in their ventricular size. Revision of the shunt confirmed obstruction at the sites indicated by infusion, and also confirmed patent opposite end. ICP intracranial pressure, HR heart rate, AMP amplitude of ICP, RAP compensatory reserve index

Analytically, testing for underdrainage relies on the calculation of mainly the critical shunt pressure, with the baseline pressure very occasionally being elevated in relationship to the shunt operating pressure. The critical shunt pressure is calculated based on the shunt resistance, infusion rate, operating pressure and average normal abdominal pressure of 5 mmHg as critical ICP (in mmHg) ≤ resistance of shunt × infusion rate + shunt operating pressure + 5 (abdominal pressure). An illustrative example of underdrainage is shown in Fig. 1a**.** In relationship to overdrainage, this can easily be assessed by testing the siphoning effect of the shunt when the patient is sitting upright, as well as assessing the difference between baseline ICP and ICP after performing the tilt test (upright position for at least 10 min until stabilisation of pressure, then flat position again). A pictorial depiction of this is shown in Fig. 1b**.** When the plateau pressure during infusion of liquid and the shunt resistance are significantly higher than the manufactured properties, combined with a higher than the manufactured resistance and excluding increased intra-abdominal pressure, a distal obstruction (valve and/or distal catheter) can be suspected. The baseline ICP is usually normal, especially in cases of chronic hydrocephalus. Occasionally, and depending on the aetiology and acuity of hydrocephalus, the baseline ICP can be significantly raised to levels too high for a functioning shunt, and a distal obstruction can be confirmed right away. Representative examples of such distal obstructions are shown in Fig. 2a, b. A ventricular catheter obstruction is accordingly observed in the absence of a detectable ICP pulse waveform when accessing the ventricular space through insertion of needles to the reservoir or prechamber. A classic example of proximal obstruction is shown in Fig. 2c. Analysis can be highly diagnostic and may also suggest partial obstruction, slit ventricles, etc. [6–9, 11, 15, 23, 26].

### Follow-up and outcome assessment

We carefully inspected all follow-up documentation from the patients’ records through our local electronic medical records (e-MR) and Epic softwares up to 6 months as well as up to 12 months from the time of the test. Searching included all follow-up clinics, possible readmissions, revision surgeries, inpatient notes if admitted and all reasons for the above. Based on the documented clinical progress and consultant impression, and mainly on the basis of relief of symptoms with good reported quality of life at follow-up, patients were marked as improving or non-improving.

## Financial analysis

We obtained billing and tariff data from our hospital’s finance department. For each shunt infusion study and shunt revision, the hospital invoices the Cambridge and Peterborough Clinical Commissioning Group (CCG) according to a fixed tariff that has been refined over the years, locally for CSF infusion tests and nationally for shunt revision operations.

All the calculations were made in pounds sterling (GBP).

CSF shunt infusion studies have been the Neurosurgery Division’s standard operating procedure for over 25 years. Hence, it was not possible to estimate, in the absence of day case CSF infusion studies, how many patients would have otherwise been managed and followed up. Therefore, in order to assess any savings achieved through infusion studies, we have assumed that the symptoms of all the patients would have been sufficiently severe to require admission either for a revision or for observation and/or ICP monitoring. Some of the patients who would have been admitted for observation and/or ICP monitoring would have required subsequent shunt revision so that the total cost of their inpatient management would have been the cost of admission, ICP monitoring AND a shunt revision.

Based on the above, we designed decision trees comparing patient flow and costs in a general neurosurgical protocol with versus without infusion studies. Data for the decision tree analysis were extracted from the national cost referencing forms 2017/18.

## Results

### Patient demographics and overall characteristics

Patients with various forms of hydrocephalus are discussed separately from patients with PTCS (including IIH) in the outcomes analysis to reflect differences in their clinical course and management.

- Hydrocephalus patient group (280 tests in 210 patients): mean age was 45 years (range 4 months to 90 years of age) with a male to female ratio of 0.84 (93 males /207 females). Forty-seven were paediatric cases (under 16 years of age). Overall, more than half of the tests (55%, 155 out of 280) had concluded there was no indication of shunt malfunction whereas 125 tests underpinned a possible malfunction (over-/underdrainage or blockage).
- PTCS group: 56 patients, 55 females, 1 male, aged 10 to 77 years old. Their CSF test results were as follows: 85 tests, 47 with possible problems and 38 without any indication for shunt malfunction. Some patients required more than 1 study within the selected period, therefore from the 56 studies 12 of them had > 1 test.

## Outcome for hydrocephalus patients with normal-functioning shunt

**Fig. 3:**
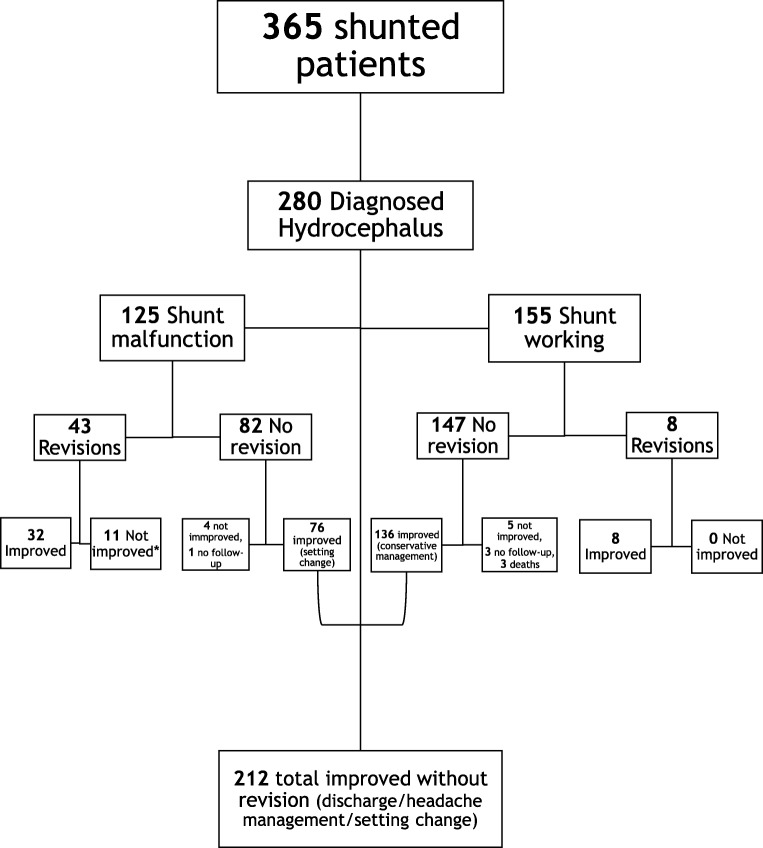
1-year outcome of patients with diagnosed hydrocephalus of multiple aetiologies undergoing CSF infusion studies for shunt function assessment in vivo. *1: Not improved after revision: One patient came back with new blockage confirmed with infusion study, but improved after the second revision. One had a wound breakdown with CSF leak and improved after system and wound revision. Another patient developed significant scarring with cheloids that required revision. Three more patients remained quite unwell, with long-term ongoing investigations between neurology and neurosurgery. Two more were discussed in MDT meeting due to some osseous and venous lesions in further imaging. One deteriorated neurologically but the deterioration was most likely due to a spinal cord syrinx, but further follow-up is not available yet. A NPH patient with complicated postshunting history never recovered and was placed in a nursing home. Finally, there was a very complex patient with very prolonged hospital stay that required multiple revisions and eventually died after years of intermittent, very long hospitalisations and very heavy problems related to her hydrocephalus

Overall, the outcomes for hydrocephalic patients are summarised in Fig. 3. Twenty-four of the patients are duplicated in the malfunctioning and functioning group, due to separate assessments over 12 months apart, showing different results, but are discussed as separate cases.

### 6-Month outcome

13/155 tests were linked to further care needs postinfusion: 4 patients required revision before a 6-month follow-up period. Three out of these 4 were most probably independent of the time and the results of the study. They required revisions because of an accident that exposed their shunt tubing (one case) or because of distal catheter migration in the other two patients a few months after the test. Three patients were lost in follow-up and three more died of causes that could not be associated with intracranial hypertension or shunt-related complications; these were for each of the three: hip fracture, recurrence of aneurysmal bleeding and a complex epilepsy syndrome. Lastly, three patients with a most likely patent shunt had ongoing symptoms and were referred for discussion in the Multidisciplinary Team meeting.

142/155 tests resulted in cases that were either discharged from neurosurgical care with instructions or referred for headache management. In some occasions, they had their shunt setting adjusted and did not require any further neurosurgical intervention. Five of these patients needed a longer period of supervision and management, mostly trying to adjust their shunts to a setting that relieved their symptoms but were managed successfully without surgery. Two patients remained unwell; one had never been well before or after shunting for NPH and had already previously suffered from severe overdrainage, requiring evacuation of bilateral subdural haematomas. The other patient appeared to suffer from refractory headaches despite attempts of adjusting their shunt setting; however, they were still managed in an outpatient basis.

Overall, 140 of the tests revealed no evident shunt malfunction on 109 unique hydrocephalic patients (27 individuals had 2 and 2 patients had 3 tests, each negative) who improved with conservative management at 6 months of follow-up.

### 12-Month outcome

Similarly, 136 cases out of the 140 that had done well at 6 months remained well after for at least 12 months, with the exception of 3 patients who required revision within 12–14 months after their initial infusion test, and one who required a revision after approximately 7 months (the single patient with refractory headaches that could not improve at 6 months by setting manipulation). Three more patients were referred for a new infusion test that showed a problem with the shunt that required changing of the setting and no revision, their follow-up records showing improvement.

The reasons for revision in all these patients are illustrated in Table 1.

**Table 1:** Reasons for revision

Reason for revision	Functioning shunts (total = 8)	Malfunctioning shunts (total = 43)
Accident	1	0
Distal catheter migration	2	0
Overdrainage	1	6
Underdrainage	4	3
Obstruction	0	34

Reasons for revision in the 8 patients with infusion test not indicating shunt failure, who required revision within a year of the test. One patient had an accident that exposed the shunt tubing, in 2 patients the distal catheter migrated and one patient was clinically diagnosed with overdrainage and had an antisyphon device only inserted. Four people were not improving with conservative management (turning down their setting) and further investigations showed underdrainage, with the neurosurgeon selecting to proceed with a revision. Reasons for revision in the group with evidence of shunt malfunction: 34 were performed due to evidence of proximal or distal obstruction after clinical review and decision. Six patients had evident overdrainage that required an antisyphon device (2 cases) or even removal of the entire system (4 cases, due to desire to change the fixed valves with programmable ones)

## Outcome of hydrocephalus patients with evidence of shunt malfunction

In 125 cases (112 unique patients, 13 had 2 tests, each showing malfunction), the infusion test detected a problem with the shunt’s function. Those problems were underdrainage, over drainage or distal/proximal blockage.

Analytically, there were 27 reports of underdrainage, 33 of over drainage, 48 blocked shunts, 8 with some disturbance of CSF dynamics (e.g. increased resistance), 1 elevated abdominal pressure, 2 slit ventricles (obstructing the proximal catheter but resolved after infusion) and 6 with no clear distinction between two possible problems, 3 with either over drainage or blockage, 1 with either slit ventricles or blockage and 2 with burr hole valves in situ which makes the interpretation of the results difficult due to the construction of the device [6].

### Outcome after revision—6 and 12 months

Overall, 43 revision operations were performed in patients with evidence of obstruction on infusion studies; 2 revisions as well as 2 infusions were performed in 2 patients who required revision and their new shunts blocked shortly after surgery. For 33 of them, the infusion study had indicated blockage (proximal, distal or both catheters), 8 underdrainage and 2 overdrainage.

Thirty-two improved sustainably at 6 months and even after 1 year, 5 by changing their shunt setting in the meantime. For some of the rest, it could be possible that their setting was changed but it was not noted in our records and some of them were discharged or referred to neurology.

The remaining 11 patients had no change in their symptoms, with persisting headaches dominating in all of them. Six had to be discussed in our MDT meeting for possible styloidectomy, stenting, etc. Unfortunately, 5 showed no improvement even after their shunt setting was adjusted postsurgically (for a long time of follow-up, 6–12 months, even after neurology referral).

The reasons for revision in this group of patients are illustrated in Table 1.

### Outcome of patients with shunt malfunction but without revision

Of the other 82/125, 1 was lost in follow-up. Four patients were classified as non-improving. Two presented with new episodes of seizures and had to be managed with antiepileptics and the other 2 were discussed in a MDT meeting ([16, 24]).

Seventy-seven non-revised patients with conservative management (setting manipulation or discharge/neurology referral) had improvement or acceptable control of their symptoms and did not require additional care for at least 1 year after their initial encounter for infusion studies. Two of these cases had evidence of proximal shunt obstruction but clinical indications of improvement soon after the infusion study, sustained for at least a year, indicating resolution of the obstruction, most likely from flushing the proximal catheter during infusion. One also had radiological evidence of significantly smaller ventricles.

From the 81/82 with non-obstructive shunt malfunction and available follow-up and the 149/155 with normal shunt function available for follow-up, a total of 142 + 77 = 219/230 clinically showed that they did not require revision and were symptom-free. This translates to a 95.22% negative predictive value (NPV) of shunt testing in vivo for excluding shunt obstruction.

## Outcome of PTCS patients

Very few PTCS patients’ shunts were deemed as blocked after an infusion study. Those patients have complicated courses and are often not only considered for revision but also for stenting, styloidectomy, temporal decompressions, etc., so we assessed them differently, according to our aim: regardless of whether a problem was detected (over-/underdrainage), we found that 46 of the patients with patent shunts were successfully managed with no neurosurgical intervention. The conservative management provided was the same as that for our hydrocephalus patients and are mentioned above extensively. These patients likewise remained well with no further care needs for at least 1 year of follow-up.

Twelve tests led to a revision shortly afterwards, after which 11 of them improved.

The 27 other patients investigated were found either as non-improving, requiring multiple revisions or different surgeries (usually venous stenting) and closer medical attention and discussions.

**Fig. 4:**
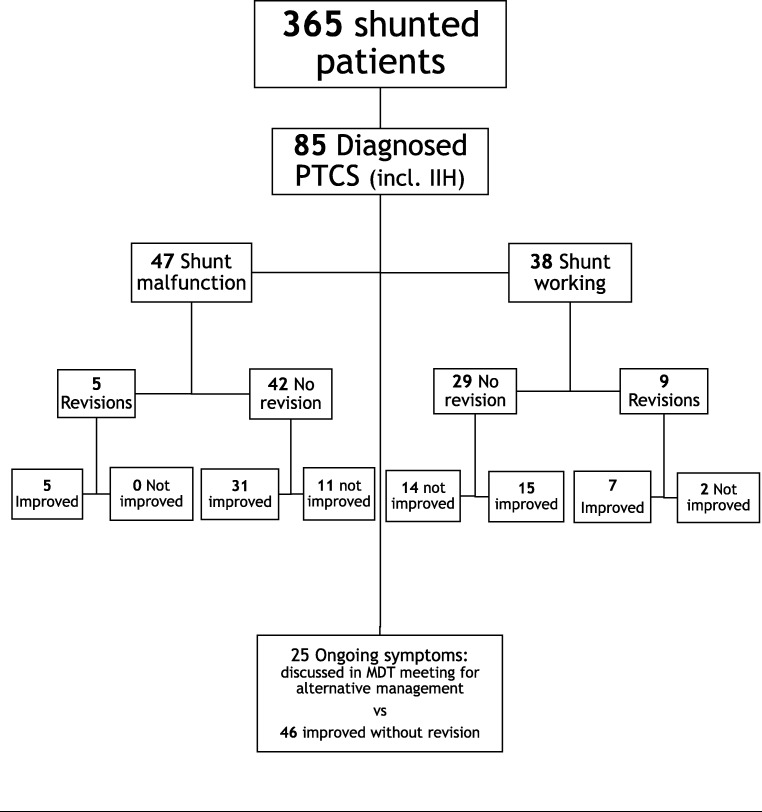
1-year outcome of patients with diagnosed pseudotumour cerebri syndrome undergoing CSF infusion studies for shunt function assessment in vivo

The overall outcome of PTCS patients is illustrated in Fig. 4**.**

### Further investigations and complications

Four hydrocephalus patients were admitted electively after the infusion test for overnight ICP monitoring. Two of the monitoring sessions confirmed overdrainage (there was suspicion but not strong evidence of overdrainage in the infusion test) and two required assessment of ICP and it dynamics for clinical and safety reasons, because a proximal obstruction did not allow reading and recording of the ICP during the infusion test.

Five PTCS patients were referred for ICP monitoring due to unresolving symptoms suspicious of active disease.

Our infection rate both for acute meningitis and chronic, subacute infections was 0%. This was assessed through patients’ medical records and revision requirements. All infections and CSF samples related to shunts are referred to Cambridge regardless of local area, from all of our catchment area on East of England.

## Health economics analysis

The current financial analysis does not separate the hydrocephalus and PTCS groups, because there were no significant differences in their care costs related to shunt problems.

### Cost of and income received for shunt reservoir infusion studies

The cost of day case admission for patients with possible shunt malfunction were calculated by the Trust’s Finance Department and included:

- Transport expenses,
- Staff: nursing, medical physics and medical staff involved,
- Cost of the shunt infusion studies equipment (including medical equipment for shunt tapping and for the computerised infusion study).

The total cost of each reservoir infusion study to the Trust was 844 GBP that matched the exact income received.

### Tariff for a shunt revision

Each shunt revision is billed to reflect

- Anaesthetic and theatre time,
- The medical and nursing staff involved,
- Theatre equipment and consumables including the cost of the valve and shunt catheters,
- Total length of hospital stay, and
- The management of any postoperative complications.

The total cost of a shunt revision procedure ranged from 9437 to 12,436 GBP (average of 10,937 GBP). The wide range in costing is due to different comorbidities that affected the cost of the surgery, as well as perioperative complications, care needs and length of stay.

### Overall financial benefit of CSF infusion studies

From our outcome analysis data, it can be extrapolated that

- The total cost of 365 CSF infusion studies was £307,695 (365 × 843).
- The actual number and cost of shunt revision operations was 65x£10,937 = £710,905.
- The total cost to the Trust of 365 studies plus actual number of Shunt revisions = £1,007,663.

**Fig. 5:**
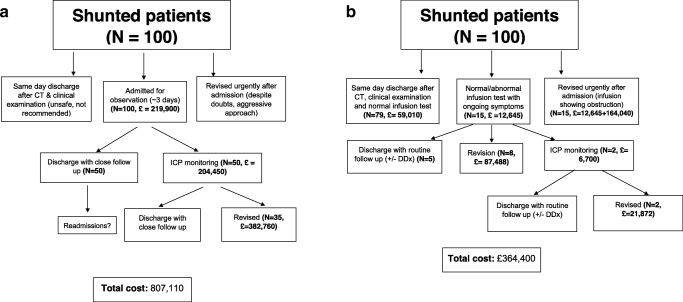
Elementary decision tree analysis of **a** costs of shunt malfunction management without infusion studies, **b** costs of shunt malfunction management as derived from our infusion study patients. Data derived from national reference costs 2017/18 and hospital income/outcome records. On average, as derived from our dataset in Table 1, around 35% of possible shunt malfunctions are due to shunt obstruction or are not amendable with shunt reprogramming. Around half of the patients do not require revision, with good resolution of symptoms. This rationale was used to calculate costs of managing patients without shunt infusions. The benefit of diagnostic information derived by infusions allows routine instead of close monitoring and facilitation of differential diagnosis of symptoms. The cost of saving follow-ups could not be approximated with the current design and dataset from our hospital. Furthermore, panel **a** cannot approximate the cost of extra hospital days in those receiving overnight monitoring with or without revision, as it is not common or standard practice in our centre

In respect of what might have happened in the absence of a CSF infusion test service, two scenarios have been explored: first, that all patients would have had a shunt revision in the absence of a CSF infusion study, OR second, that a proportion of patients admitted for observation would have had ICP monitoring, 70% of whom would have gone on to shunt revision (decision trees—Fig. 5a, b).

Scenario 1

(d) The cost of shunt revision operations avoided was 299 (Figs. 3 and 4 sum of all non-revised) × £10,937 = £3,270,163.
(e) The total cost to the Trust if no CSF infusion study service = ~ 3,992,005 (365 × 10,937)

Scenario 2

Therefore, from all of the above, the total saving to the Trust by providing a CSF infusion service = ~ £2,766,081 over 3 years = ~ £922,027 per annum.

When comparing infusion studies to a standard protocol, using only ICP monitoring and exploratory surgery, the overall financial benefit could be approximately £442,710 per 100 patients admitted with possible shunt malfunction. An analytical decision tree showing a comparative cost analysis between using infusion studies versus no infusion studies is presented in Fig. 5**.**

## Discussion

This study provides the first long-term, clinical outcome-orientated evidence to support the importance of avoiding revisions of patent shunts and optimizing shunt diagnostics through the incorporation of infusion studies in every day neurosurgical practice, as at the Regional Neurosurgery Unit at Addenbrooke’s Hospital, Cambridge (catchment population ~ 3 million). Our large cohort of patients, complication-free method and catchment population could translate to a potentially high external validity and applications of our findings in healthcare systems worldwide.

There were three main findings in our analysis:

1. The use of infusion studies as a supportive tool for clinical decisions on shunt revision helped in avoiding over 290 (299 long-term at 12 months) unnecessary operations over the course of 3 years.
2. 258—over 200 and around 86%—of those patients non-surgically managed remained well long-term without persisting symptoms or additional care requirements. This translates to a very high negative predictive value of the shunt infusion test, a point which should be investigated independently in a separate study.
3. A significant amount of money was saved and the burden on patients of unnecessary shunt revision operations averted.

Importantly, patients with normally functioning shunts remained well after discharge or under pharmacological care that was sufficient for their headaches. Not a single patient presented with acute/severe problems after being discharged and no patient’s shunt was infected as a result of the test. It is our hospital’s policy to remove shunt systems if there is a CNS infection. If any of the above patients had an infection attributed to their shunt infusion test, they would have been referred to the neurosurgical team for review and management.

Infusion tests provide reliable evidence that the shunt is not obstructed and does not allow ICP to exceed a desired range [3, 6, 10, 14]. Patients can therefore be discharged home safely and booked for a routine follow-up that will allow more time and better planning to be invested into their review and care.

### Hydrocephalus patients

Infusion tests appeared quite accurate and efficient in directing away from shunt malfunction in hydrocephalus, as demonstrated by the 212 cases that were managed conservatively without further hospitalisations. Shunt testing in vivo appears to be most valuable to our selected cases that did not have a radiological signs substantiative of shunt malfunction and were not acutely unwell. This can often be the case with NPH patients, as well as younger patients with chronic or neglected hydrocephalus [2, 6, 8, 22, 23, 25, 43].

It has been highlighted in multiple reports of paediatric hydrocephalus patients that an increased number of revision operations is a negative predictive factor for cognitive outcome and overall disability and quality of life for paediatric patients [3–6, 9, 27, 28]. For adults, this increased number also is a predictor of further shunt revision surgery requirements in the future and therefore constant care needs, many hospitalisations and reduced quality of life [3, 6, 17, 29]. Importantly, over 85% of our patients lead a symptom-free and neurosurgically uneventful life for at least 1 year of follow-up. Furthermore, patients were spared the consequences of longer hospital stay and the additional complications of unnecessary shunt revision surgery, whose rates and sequelae have been reported extensively in the literature [18, 19, 27].

### PTCS patients

The pathophysiology of PTCS is complex and often involves concomitant elevation of the venous sinus pressures and CSF pressures that appeared to be coupled [12, 33, 34, 40]. However, a shunt infusion could be useful in pointing towards or away from a shunt problem, facilitating patient flow and referral for stenting or other management. There were 15 patients with proven working shunts on their CSF infusion studies whose symptoms could not be relieved with conservative management. They were referred back to the PTCS multidisciplinary team for further investigations and consideration of alternative treatments without being subjected to a revision that would most likely not have benefitted them and would have delayed their referral to a more appropriate service.

### Predictive value of the shunt infusion test

Based on the data from symptom relief and follow-up alone, the NPV of shunt infusion tests was 95%. This refers to the accuracy with which a shunt infusion test can exclude a shunt obstruction. In order to accurately reflect the positive predictive value as well as the NPV of the test, the results from the intraoperative shunt flow testing should be compared with the infusion test result. As our patients with a negative (exclusive of obstruction) shunt infusion test would not undergo a revision in their majority, it would not be possible to calculate this in our current dataset. Furthermore, as from our 51 revised patients, not all the intraoperative notes clearly indicated the site of obstruction; the low number of patients currently tested would not be able to yield a reliable positive predictive value. Finally, we only calculated the NPV derived from hydrocephalus patients, whose clinical course as mentioned is more straightforward, whereas PTCS patients present with multidisciplinary challenges that confound their assessment based on shunt patency alone. A new design, aiming to test this diagnostic accuracy with a high number of revised patients, should be performed to address this.

### Financial impact of avoiding shunt revisions on the NHS

Overall, the surgical management of CSF disorders is very cost-effective in comparison with the rest of medicine in general. The average cost per QALY was only £215 in 1990 in comparison, for example, to £750 for a hip replacement or £700 for a pacemaker. Length of stay has reduced considerably since 1990 so that the cost per QALY will have reduced further in relative terms. Antibiotic-impregnated shunt catheters have been shown to be cost-effective with cost savings of $42,125 and $230,390 per 100 de novo shunts placed in adult and paediatric patients respectively [35].

As expected, avoiding surgical revisions in shunted patients seems to be of considerable financial benefit to the NHS. However, as stated in the methods, it is not possible to know exactly how many of these patients would have been selected for shunt revision, if infusion studies were not available. There are other methods that are used worldwide in order to diagnose shunt malfunction. Overnight ICP monitoring remains the gold standard, but it is also quite invasive, involves cranial surgery and has its own cost implications. Other methods, less invasive, involve radiation (such as radio-contrast shuntograms, radioactive flow studies) or no radiation (MRI/high resolution MRI, optic sheath diameter measurements, etc.). However, they all implicate the cost of a radiologist or other highly specialised clinical staff, expose the patient to radiation and even endanger the functionality and/or patency of the shunt [2, 4, 6, 10, 23]. In addition, measurements of steady-state ICP cannot exclude shunt malfunction. No flow through a shunt does not automatically mean obstruction, but many other things, including inadequate pressure to open the valve or even collapsed ventricles (slit ventricles) around the proximal catheter, among others [4, 6, 10, 23, 28, 29, 32, 43]. Last but not least, our current protocol for performing shunt infusion studies that includes strict aseptic technique and proper cleaning and disinfection appears to be 100% effective in avoiding additional costs related to infections from the procedure.

Shunt revision is a predictive factor for multiple future revisions; therefore, averting one redundant revision translates to averting multiple revisions in the long-term [3, 4, 7–9, 39]. A recent report from the UK shunt registry has indicated the revision rates in all UK centres, where our centre holds a lower revision rate compared to the average, especially after first implantation [41]. Another aspect considered in the revision costs, is that the comprehensive nature of infusion studies allows the clinical team to be aware, and not blinded to, the location of the shunt issue. A proximal catheter obstruction can easily be differentiated from a valve or distal catheter problem [6, 7, 10, 11, 23, 26, 28, 32, 38]. This most probably decreases the cost of the revision surgery, since parts of the shunt can be left intact, decreasing surgical time, use of equipment, complications and postoperative hospital stay. However, investigating this was not within the scope of our current paper and could be the subject of a different study.

## Limitations

The main limitation of this study is that it is from a single centre and it was unknown how many patients would have had a shunt revision in the absence of a CSF infusion study. We have estimated the costs based on a 50% rate of ICP monitoring and 70% rate of revision in this subgroup of patients (Fig. 4a). A shunt infusion study requires access to a prechamber. This shunt anatomy is present in most valves in the market, with the exception of the fixed-pressure burr hole valve [6].

## Electronic supplementary material

ESM 1 (PDF 123 kb)

## Abbreviations

CSF: Cerebrospinal fluid
IIH: Idiopathic intracranial hypertension
PTCS: Pseudotumour cerebri syndrome
CT: Computed tomography
MRI: Magnetic resonance imaging
e-MR: Electronic medical record

## References

[1] Antes S, Stadie A, Müller S, Linsler S, Breuskin D, Oertel J (2018) Intracranial Pressure–Guided Shunt Valve Adjustments with the Miethke Sensor Reservoir. World Neurosur 642–650 10.1016/j.wneu.2017.10.044 29054776

[2] Arrington CN, Ware AL, Ahmed Y, Kulesz PA, Dennis M, Fletcher JM (2016). Are shunt revisions associated with IQ in congenital hydrocephalus? a meta -analysis. Neuropsychol Rev.

[3] Aylward SC, Reem RE (2017). Pediatric neurology pediatric intracranial hypertension. Pediatr Neurol.

[4] Boyle TP, Nigrovic LE (2015). Radiographic evaluation of pediatric cerebrospinal fluid shunt malfunction in the emergency setting. Pediatr Emerg Care.

[5] Bromby A, Czosnyka Z, Allin D, Richards HK, Pickard JD, Czosnyka M (2007). Laboratory study on “intracranial hypotension” created by pumping the chamber of a hydrocephalus shunt. Cerebrospinal Fluid Res.

[6] Chari A, Czosnyka M, Richards HK, Pickard JD, Czosnyka ZH (2014). Hydrocephalus shunt technology: 20 years of experience from the Cambridge Shunt Evaluation Laboratory. J Neurosurg.

[7] Czosnyka M (1996) Testing of cerebrospinal compensatory reserve in shunted and non-shunted patients: a quide to interpretation based on an observationl study. J Neurol Neurosurg Psychiatry c(November 1995):549–558 10.1136/jnnp.60.5.549 PMC486369 8778261

[8] Czosnyka M, Pickard JD (2004). Monitoring and interpretation of intracranial pressure. J Neurol Neurosurg Psychiatry.

[9] Czosnyka M, Whitehouse H, Smielewski P, Simac S, Pickard JD (1996) Testing of cerebrospinal compensatory reserve in shunted and non-shunted patients: a guide to interpretation based on an observational study. J Neurol Neurosurg Psychiatry. 10.1136/jnnp.60.5.549 10.1136/jnnp.60.5.549 PMC486369 8778261

[10] Czosnyka M, Czosnyka ZH, Momjian S, Pickard JD (2004). Cerebrospinal fluid dynamics. Physiol Meas.

[11] Czosnyka Z, Czosnyka M, Owler B, Momjian S, Kasprowicz M, Schmidt EA, Smielewski P, Pickard JD (2005) Clinical testing of CSF circulation in hydrocephalus. Acta Neurochir Suppl c(95):247–251 10.1007/3-211-32318-x_50 16463858

[12] Czosnyka Z, Czosnyka M, Owler B, Momjian S, Kasprowicz M, Schmidt EA, Smielewski P, Pickard JD (2005). Clinical testing of CSF circulation in hydrocephalus. Acta Neurochir Suppl.

[13] Czosnyka M, Czosnyka Z, Agarwal-harding KJ, Pickard JD (2012). Modeling of CSF dynamics: legacy of Professor Anthony Marmarou. Acta Neurochir Suppl.

[14] Czosnyka M, Czosnyka Z, Agarwal-harding KJ, Pickard JD (2012) Modeling of cerebrospinal fluid dynamics: legacy of Professor Anthony Marmarou. doi: 10.1007/978-3-7091-0923-6 10.1007/978-3-7091-0923-6_2 22116414

[15] Czosnyka M, Czosnyka Z, Agarwal-harding KJ, Pickard JD (2012) Hydrocephalus. doi: 10.1007/978-3-7091-0923-6 10.1007/978-3-7091-0923-6_2 22116414

[16] Dashti SR, Nakaji P, Hu YC, Frei DF, Abla AA, Yao T, Fiorella D (2012). Styloidogenic jugular venous compression syndrome: diagnosis and treatment: case report. Neurosurgery.

[17] Dias SF, Lalou A, Spang R, Haas-Lude K, Garnett M, Fernandez H, Czosnyka M, Schuhmann MU, Czosnyka Z (2019) Value of computerized shunt infusion study in assessment of pediatric hydrocephalus shunt function—a two center cross-sectional study. Childs Nerv Syst. 10.1007/s00381-019-04264-3 10.1007/s00381-019-04264-3 31372736

[18] Dupepe EB, Hopson B, Johnston JM, Rozzelle CJ, Oakes WJ, Blount JP, Rocque BG (2016). Rate of shunt revision as a function of age in patients with shunted hydrocephalus due to myelomeningocele. Neurosurg Focus.

[19] Edwards NC, Engelhart L, Casamento EMH, McGirt MJ (2015). Cost-consequence analysis of antibiotic-impregnated shunts and external ventricular drains in hydrocephalus. J Neurosurg.

[20] Ekstedt J (1977). CSF hydrodynamic studies in man. 1. Method of constant pressure CSF infusion. J Neurol Neurosurg Psychiatry.

[21] Ekstedt J (1978). CSF hydrodynamic studies in man 2 normal hydrodynamic variables related to CSF pressure and flow. J Neurol Psychiatry.

[22] Goeser CD, McLeary MS, Young LW (1998). Diagnostic imaging of ventriculoperitoneal shunt malfunctions and complications. RadioGraphics.

[23] Hart MG, Czosnyka M, Czosnyka ZH, Fernandes HM (2014) Combined intracranial pressure monitoring and cerebrospinal fluid infusion study to guide management of slit ventricle syndrome. Pediatr Neurosurg. 10.1159/000358561 10.1159/000358561 24525521

[24] Higgins JN, Garnett MR, Pickard JD, Axon PR (2017). An evaluation of styloidectomy as an adjunct or alternative to jugular stenting in idiopathic intracranial hypertension and disturbances of cranial venous outflow. J Neurol Surg B.

[25] Jorgensen J, Williams C, Sarang-Sieminski A (2016). Hydrocephalus and ventriculoperitoneal shunts: modes of failure and opportunities for improvement. Crit Rev Biomed Eng.

[26] Kim D-J, Kim H, Kim Y-T, Yoon BC, Czosnyka Z, Park K-W, Czosnyka M (2015) Thresholds of resistance to CSF outflow in predicting shunt responsiveness. Neurol Res. 10.1179/1743132814Y.0000000454 10.1179/1743132814Y.0000000454 25323618

[27] Korinek AM, Fulla-Oller L, Boch AL, Golmard JL, Hadiji B, Puybasset L (2011). Morbidity of ventricular cerebrospinal fluid shunt surgery in adults: an 8-year study. Neurosurgery.

[28] Lavinio A, Czosnyka Z, Czosnyka M (2008). Cerebrospinal fluid dynamics. Eur J Anaesthesiol.

[29] Limbrick DD, Baird LC, Klimo P, Riva-Cambrin J, Flannery AM (2014). Pediatric hydrocephalus: systematic literature review and evidence-based guidelines. Part 4: cerebrospinal fluid shunt or endoscopic third ventriculostomy for the treatment of hydrocephalus in children. J Neurosurg Pediatr.

[30] Lo TYM, Myles LM, Minns R (2003) Long-term risks and benefits of a separate CSF access device with ventriculoperitoneal shunting in childhood hydrocephalus. Dev Med Child Neurol:28–33 12549752

[31] Lofgren JAN, Zwetnow N (1973) The pressure-volume curve of the cerebrospinal fluid space in dogs. Acta Neurol Scand C:557–574 10.1111/j.1600-0404.1973.tb01330.x 4770652

[32] Nabbanja E, Pickard JD, Lalou AD, Czosnyka ZH (2018) Use of CSF infusion studies to unblock occluded hydrocephalus ventricular shunt catheters: a preliminary report of two patients. BMJ Case Rep bcr-2017-223861 10.1136/bcr-2017-223861 PMC5965807 29769186

[33] Owler BK, Parker G, Halmagyi GM, Dunne VG, Grinnell V, McDowell D, Besser M (2003). Pseudotumor cerebri syndrome: venous sinus obstruction and its treatment with stent placement. J Neurosurg.

[34] Owler BK, Parker G, Halmagyi GM, Johnston IH, Besser M, Pickard JD, Higgins J (2005). Cranial venous outflow obstruction and psudotumor cerebri syndrome. Adv Tech Stand Neurosurg.

[35] Parker SL, Mcgirt MJ, Murphy JA, Megerian JT, Stout M, Engelhart L (2014). Cost savings associated with antibiotic-impregnated shunt catheters in the treatment of adult and pediatric hydrocephalus. World Neurosurg.

[36] Paulsen AH, Lundar T, Lindegaard KF (2015). Pediatric hydrocephalus: 40-year outcomes in 128 hydrocephalic patients treated with shunts during childhood. Assessment of surgical outcome, work participation, and health-related quality of life. J Neurosurgery-Pediatrics.

[37] Petrella G, Czosnyka M, Keong N, Pickard JD, Czosnyka Z (2008). How does CSF dynamics change after shunting?. Acta Neurol Scand.

[38] Petrella G, Czosnyka M, Smielewski P, Allin D, Guazzo EP, Pickard JD, Czosnyka ZH (2009). In vivo assessment of hydrocephalus shunt. Acta Neurol Scand.

[39] Pickard J, Bailey S, Sanderson H, Rees M, Garfield JS (1990). Steps towards cost-benefit analysis of regional neurosurgical care. BMJ.

[40] Pickard JD, Czosnyka Z, Czosnyka M, Owler B, Higgins JN (2009) Coupling of sagittal sinus pressure and cerebrospinal fluid pressure in idiopathic intracranial hypertension – a preliminary report. Acta Neurochir Suppl:283–285 10.1007/978-3-211-85578-2_53 19388330

[41] Pickard JD, Richards H, Joannides A (2017) UK Shunt Registry Draft Report:2017

[42] Richards H, Seeley H, Pickard J (2009). Who should perform shunt surgery? Data from the UK Shunt Registry. Cerebrospinal Fluid Res.

[43] Schuhmann MU, Sood S, McAllister JP, Jaeger M, Ham SD, Czosnyka Z, Czosnyka M (2008) Value of overnight monitoring of intracranial pressure in hydrocephalic children. Pediatr Neurosurg. 10.1159/000131675 10.1159/000131675 18480615

[44] Spiegelman L, Asija R, Da Silva SL, Krieger MD, McComb JG (2014). What is the risk of infecting a cerebrospinal fluid–diverting shunt with percutaneous tapping?. J Neurosurg Pediatr.

[45] Spirig JM, Frank MN, Regli L, Stieglitz LH (2017) Shunt age-related complications in adult patients with suspected shunt dysfunction . A recommended diagnostic workup. 1421–1428 10.1007/s00701-017-3237-6 28616668

[46] Tamber MS, Klimo P, Mazzola CA, Flannery AM (2014). Pediatric hydrocephalus: systematic literature review and evidence-based guidelines. Part 8: management of cerebrospinal fluid shunt infection. J Neurosurg Pediatr.

[47] Weerakkody RA, Czosnyka M, Schuhmann MU, Schmidt E, Keong N, Santarius T, Pickard JD, Czosnyka Z (2011). Clinical assessment of cerebrospinal fluid dynamics in hydrocephalus. Guide to interpretation based on observational study. Acta Neurol Scand.

